# Advances and challenges in genetic technologies to produce single-sex litters

**DOI:** 10.1371/journal.pgen.1008898

**Published:** 2020-07-23

**Authors:** Charlotte Douglas, James M. A. Turner

**Affiliations:** Sex Chromosome Biology Laboratory, The Francis Crick Institute, London, United Kingdom; University of Michigan Medical School, UNITED STATES

## Abstract

There is currently a requirement for single-sex litters for many applications, including agriculture, pest control, and reducing animal culling in line with the 3Rs principles: Reduction, Replacement, and Refinement. The advent of CRISPR/Cas9 genome editing presents a new opportunity with which to potentially generate all-female or all-male litters. We review some of the historical nongenetic strategies employed to generate single-sex litters and investigate how genetic and genome editing techniques are currently being used to produce all-male or all-female progeny. Lastly, we speculate on future technologies for generating single-sex litters and the possible associated challenges.

## Introduction

Animal models remain indispensable experimental reagents for understanding fundamental biology and translational research. Despite this utility, there is an ongoing issue with the production of animals that are surplus to requirement. For example, in 2017, in Great Britain alone, over 1.8 million laboratory animals were culled without ever being used for a scientific procedure [1]. Globally, the Reduction, Replacement, and Refinement (3Rs) principles are common factors that encourage the reduction of unnecessary animal use [2]. For example, in the European Union, evidence of adhering to the 3Rs is a legal requirement, and in the USA, the goal of the Animal Welfare Act is to encourage alternative experimental strategy to minimise animal pain and distress. One factor contributing to excess production of animals is sex-specific research; for example, studies of reproductive biology or sex-specific cancers in which only one sex is required (Table 1). A genetic method of producing single-sex litters, in which the unrequired sex is nonviable in utero and therefore is never born, would remove the need for postnatal culling, in line with the 3Rs.

**Table 1:** A summary of major examples of different species and the current requirements for single-sex litters.

Species	Sex chromosomes	Sex required	Why are single sexes required
Mosquito	XX female XY male	Males	Females carry the malaria parasite
Mouse	XX female XY male	Males	Male-specific scientific research Laboratory-controlled sterility of all-male litters for population control
Mouse		Females	Female-specific scientific research
Chicken (layers)	ZW female ZZ male	Females	Egg-laying
Cows (dairy)	XX female XY male	Females*	Milk production for dairy products
Cows (meat)		Males	Greater mass for meat
Silkworm	ZW female ZZ male	Males	Greater quality of silk production
Fruit fly	XX female XY male	Males	Laboratory-controlled sterility of all-male litters for pest population control
Insects	Variable	Males	Laboratory-controlled sterility of all-male litters for pest population control

There is often a requirement for a single sex in food production because only one sex is able to produce an animal product: for example, eggs by female layer hens. However, there is also a requirement to produce all-male litters that can be sterilised in controlled laboratory or factory conditions prior to release in the wild. In this strategy, the overall population size can be reduced for pest-control measures, such as for insects and rodents. This strategy for pest control is called SIT. **Abbreviations:** SIT, Sterile Insect Technique.

*In some countries—for example, the USA—male offspring produced in the dairy cow industry are repurposed for the meat supply chain.

The requirements for all-female or all-male litters is not limited to laboratory models (Table 1). For example, it would also be extremely advantageous for agriculture, with the layer hen industry representing a prominent example. Approximately 6 to 7 billion male chicks are culled worldwide per year, generating a well-known and highly controversial ethical issue [3]. Conversely, in pest control, reducing or controlling the female mosquito population, the vector for the malaria parasite, found in over 100 countries including large parts of Africa and Asia, would be extremely advantageous, and similarly for the eradication of invasive pest species such as rodents in island countries such as New Zealand [4]. In these examples, a genetic method of producing all-male litters in a controlled laboratory and factory environment for sterilisation, prior to release in the wild, would eliminate or reduce the population size. One alternative method of controlling malaria spread would be to repurpose engineered gene drives in order to produce single-sex progeny.

The production of all-female or all-male litters by genetic methods is feasible because in some species, males and females differ in their sex-chromosome complement (Table 1). Eutherian female mammals, such as mice and humans, are homogametic, producing only X-chromosome–carrying gametes. Eutherian male mammals are heterogametic, producing mature sperm that, with rare exceptions (for example, [5–7]), carry either the X or Y chromosome. Early studies on differences of sex determination (DSDs) showed that in eutherian mammals, sex determination is not regulated by the number of X chromosomes [8,9]. Instead, it is driven by the presence of the Y chromosome via a locus originally coined the Y-linked testis-determining factor (TDF; [10]). The TDF was later identified to be *SRY/Sry* (Sex-determining region Y), which is expressed in Sertoli cell precursors [11–17]. It is important to note that the *SRY/Sry* mode of sex determination is not the primary method of sex determination for all mammals. For example, the platypus, a prototherian mammal, does not have an *SRY* gene [18,19].

Conversely, in many bird species, including chickens, females are heterogametic and carry a single Z and a single W chromosome. Males are homogametic and carry 2 Z chromosomes. Avian sex determination is controlled by the dosage of a Z-linked gene called DMRT1 (Doublesex and mab-3–related transcription factor 1; [20]). Female birds carry 1 copy of DMRT1, whilst males have 2 copies. DMRT1 is an orthologue of *doublesex* that undergoes sex-specific alternative splicing to regulate sex determination in many insect species, including *Drosophila melanogaster*, reviewed in [21].

In this Review, we investigate some of the current requirements for single-sex litters in research and in other industries such as agriculture and pest control. We describe some of the historical methods for sexing-sorting and advantages and disadvantages associated with them. We then discuss current methods performed to generate single-sex progeny, including by genome editing methods such as CRISPR/Cas9 [22–26]. Finally, we assess the challenges associated with generating single-sex litters and future perspectives of the technologies.

## Previously developed nongenetic methods for producing single-sex litters

Historically, sex selection is performed by investigating sex-specific biological differences. For example, male and female chick embryo allantoic fluid contains differential levels of estrone sulfate [27]. However, such methods of determining sex by allantoic fluid extraction are invasive and generally give the most reliable results at day 9 of development, which is close to the onset of pain perception [3,27]. Furthermore, invasive procedures frequently result in reduced viability [28,29]. Recently, Galli and colleagues performed fluorescence and Raman spectroscopy to determine differential hormone levels in male and female chick embryonic blood at day 3.5 [30]. Although this method does not require fluid extraction, it requires a window to be made in the shell and therefore may still be considered invasive. Moreover, although accurate, this and other methods still result in culling of chick embryos during late stages of development. Therefore, the sex-selection field is now developing alternative approaches to produce single-sex litters by genetic methods, whereby the unrequired sex is eliminated at an earlier embryonic stage in utero without the need for mechanic or spectroscopic testing.

### Physical separation of mammalian X- or Y-carrying sperm

Given the risk of embryonic nonviability and pain perception associated with invasive procedures for sexing embryos, one superior method to selecting offspring sex is by separation of X- or Y-carrying sperm. Prior selectivity of sex-specific gametes for in vitro fertilisation (IVF) or artificial insemination (AI) ensures that the offspring sex is predictable, which may be more economically viable and ethically justifiable than invasive sexing procedures.

Many techniques have been previously attempted to isolate X- and Y-sperm, including fluorescence in situ hybridisation (FISH) and swim separation [31]. The most successful method for selective separation of X- or Y-carrying sperm for IVF/AI is by flow cytometry [32]. Sperm nuclei are stained using Hoechst 33342 and sorted based on DNA content. Bull X-carrying sperm, for example, have approximately 3.8% greater DNA content compared to Y-sperm [33]. The main caveat of this flow cytometric approach is that the sperm exhibit reduced fertilisation ability [34–37]. Increasing the quantity of sperm used for AI does not appear to significantly rescue the conception rate [38], suggesting that the reduced fertility results from sorting and postsorting procedures and may possibly be due to residual Hoechst dye [35,36].

Flow cytometry is associated with large economic and time costs in sperm sorting and postsort procedures (reviewed in [37]), leading to the investigation of alternative methods. One such strategy is to separate X- and Y-carrying sperm by sex-chromosome–specific differential gene expression. However, this strategy has remained extremely challenging because during spermatogenesis, the X- and Y-sperm are connected via cytoplasmic bridges. The cytoplasmic bridge connections are essential to ensure that the haploid X-carrying sperm receive Y-carrying sperm products (and vice versa), as well as mRNAs [39,40] and organelles [41]. However, an intriguing recent study by Umehara and colleagues highlighted that cell-surface marker Toll-like receptor 7/8 (TLR7/8) was expressed on X-carrying, but not Y-carrying, sperm [42]. Ligand activation of the TLR7/8 receptor using Resiquimod or Imiquimod suppressed X-sperm motility, allowing for X- and Y-carrying sperm separation prior to IVF procedures. Following IVF with the X-carrying ‘slow’ sperm, the proportion of female offspring was 81%, whilst following IVF with Y-carrying ‘fast’ sperm, the proportion of male pups was 83% [42]. Umehara and colleagues performed the work using mouse as a model but speculated that the strategy was translatable to many agricultural species.

Another technique also utilising cell-surface markers for separating X- and Y-sperm, used in bulls, is ‘WholeMom’ [43]. In this approach, a monoclonal antibody selectively binds an epitope only present on the bull Y-chromosome–carrying sperm plasma membrane. Epitope binding results in agglutination of the Y-carrying sperm heads, whilst the X-carrying sperm are unaffected and fertilise oocytes [43].

In summary, current approaches rely on the differential DNA content or surface markers of X- and Y-carrying sperm in order to physically separate the sperm. These strategies rely on IVF of sex-sorted sperm to skew offspring sex ratios. Although these and other methods not described in detail in this Review (listed in Table 2) are occasionally feasible approaches to sex selection, many are expensive, time-consuming, and often inefficient or inaccurate. Genetic approaches to sex selection are therefore being developed as alternative approaches.

**Table 2:** Nongenetic methods for sex selection.

Method	Advantages	Disadvantages	Species performed in	References
Hormone quantification	Accurate (in later developmental stages)	Invasive (affects hatching and viability) Can only be applied after day 9 (post onset of pain perception)	chicken	[27,44]
Egg shape	Noninvasive	Accuracy is variable		[45]
Egg odour	Noninvasive	Accuracy is variable		[46,47]
Raman and fluorescence spectroscopy (optical spectroscopy)	Accurate Near-infrared excitation prevents damage to cells	Invasive High background fluorescence signal		[30,48–50]
Hyperspectral imaging	Noninvasive	Limited to species with sex-specific feather colour Accuracy is variable		[51,52]
Fourier transform infrared spectroscopy	Performed on nonincubated eggs on the germinal disk	Invasive		[53]
‘Hologic Invader’	Relatively quick molecular sexing method Accurate	Has only been established in laboratory conditions		[54]
Sperm separation				
(a) Flow cytometry	High purity	Requires detectable differences in DNA size between sex chromosomes Compromised fertilisation ability of sperm High cost and time	cow, rabbit, sheep, pig	[37,55,56]
(b) Swim separation by agglutination of X- or Y-specific epitopes	No mechanical damage to the sperm	X- and Y-carrying sperm are connected by cytoplasmic bridges, controversy as to whether there is sex-specific expression	cow, buffalo, mouse	[42,43,57]
(c) Immunological assays for male-specific H-Y antigens	No mechanical damage to the sperm	Controversy as to whether the H-Y antigen is uniquely on Y-sperm	cow, mouse	[58,59]; reviewed in [60]
(d) FISH	Accurate Generally used for flow-cytometry–sorted sperm purity check	Sperm heads have to be decondensed	cow, mouse, pig, dog	[61–65]
(e) Raman spectroscopy	Efficient Noninvasive		cow	[66]
(f) Labelling with nanoparticles	Efficient for labelling	Could be toxic for sperm	cow	Reviewed in [67]
Embryo sexing				
(a) Karyotyping	Accurate Inexpensive	Difficulty in producing high quality metaphase spreads Time-consuming Embryo biopsy may affect viability		[68]
(b) Metabolomic differences	Accurate in cow embryos	Limited by the amount of quantifiable enzyme Assay may be toxic to embryos	cow	[69]
(c) Analysis of sex chromatin	Inexpensive Simple method	May not be able to detect Barr body	rabbit	[70]
(d) FISH	Low risk of contamination from other cell types. Confirmation of embryonic cell type by visualisation of FISH Highly accurate	Requires highly Y-specific probe Embryo biopsy may affect viability Time-consuming	cow	[71]
(e) H-Y antigen	Noninvasive Fairly accurate	Assay may lower embryo viability	cow, sheep, pig, horse, goat, mouse	[72]
(f) ccffDNA	Fairly accurate Noninvasive	Requires a downstream PCR analysis for sex-specific polymorphisms	sheep	[73]
Pupal size	Could be effective in small laboratory settings	Requires manual sorting Highly error-prone Species variability	insects, including mosquito	[74,75], reviewed in [76]
Behavioural differences				[77,78]

Historically, there have been many methods to attempt to produce single-sex litters. We summarise some of the main methods utilised, with the major advantages and disadvantages for each method. **Abbreviations:** ccffDNA, circulating cell free foetal DNA; FISH, fluorescence in situ hybridisation

## Current genome editing methods to generate single-sex litters

The development of genetic methods to produce single-sex litters relies on sex-specific genomic differences, such as a different sex-chromosome complement (Table 1). For example, in many insect species and eutherian males, the Y chromosome is inherited by sons, and the paternal X chromosome is inherited by the daughters. This sex-specific inheritance of the father’s sex chromosomes can be exploited in order to control the inheritance of transgenes. In many insect species, the sex-specific alternative splicing of genes such as *doublesex* can also be harnessed to ensure sex-specific expression of transgenes.

### Transgene-based sex-selection systems

Producing all-male litters would be advantageous for mosquito and insect population control. Broods of all-male litters could be generated, sterilised in controlled laboratory conditions, and then released into the wild to induce population collapse. This strategy is called the ‘Sterile Insect Technique’ (SIT; [79]). To select for males, insect species carrying sex-chromosome–specific or sex-specific fluorescent markers have been generated [80,81]. However, these strategies require manual sorting of insects.

A refinement of SIT is called ‘Release of Insects carrying a Dominant Lethal’ (RIDL; [82]). RIDL is a system of sex-specific transgene-induced lethality, thereby overcoming issues with manual sex sorting. Early successful transgenic methods for sex-specific lethality were carried out on a Lepidopteran species, *Bombyx mori* (Mulberry silkworm). Male silkworms are desirable over females because they produce higher-quality silk [83], but RIDL can also be used for generating male-only broods prior to sterilisation and release. Tan and colleagues cloned a tetracycline-repressible transactivator (tTAV) construct into an orthologous *doublesex* minigene from *Pectinophora gossypiella* (*Pgdsx*; pink bollworm) and inserted the transgene into the *B*. *mori* genome [84]. Endogenous *Pgdsx doublesex* undergoes sex-specific alternative splicing; therefore, in *B*. *mori*, tTAV expression was specific to females. The female-specific tTAV protein accumulation induced female-specific lethality, resulting in male-only cocoons surviving. Moreover, the female-specific lethality could be largely repressed by the addition of dietary tetracycline [84], allowing for control of the sex-specific lethality system. Interestingly, however, similar *doublesex*-regulated lethality constructs integrated into other pest insect genomes—for example, the olive fly and Mediterranean fruit fly—did not have the same lethality effect [85,86].

More recently, Kandul and colleagues generated an antibiotic-resistance–based sex-selection transgene system in *D*. *melanogaster* [87]. Two drug-resistance transgenes are expressed in opposite sexes by integration of each transgene into a sex-specific intron of the *transformer* or *doublesex* genes. Male and female flies have normal viability until the dietary addition of either puromycin or geneticin, which selects for males or females, respectively, producing progeny of 100% the required sex.

Although the use of fluorescence-transgene–based sexing systems were inefficient in insect species, in chickens, they are currently the most promising genetic approach for chick sexing prior to hatching. In Australia, the Commonwealth Scientific and Industrial Research Organisation (CSIRO) is championing a chick-marker approach whereby a fluorescent marker is integrated onto the male-determining Z chromosome so that male and female chicks can be segregated prior to hatching [88].

### Transgene-induced destruction of sex-specific sperm

**Fig 1:**
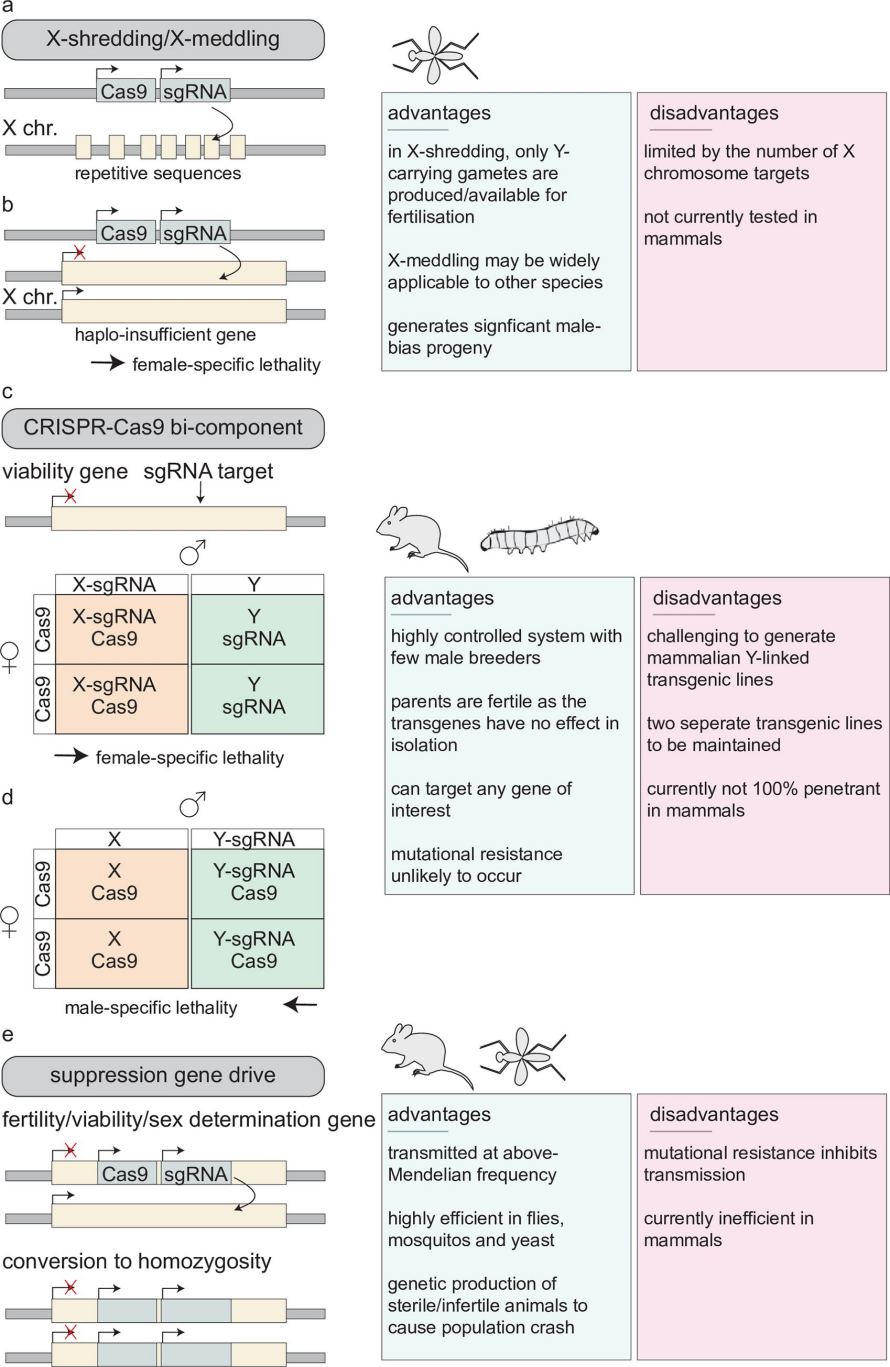
Genetic methods of producing single-sex litters. (a,b) X-shredding and X-meddling techniques are engineered to utilise CRISPR/Cas9 components to target specific regions on the X chromosome. During spermatogenesis, the CRISPR/Cas9 components are expressed and induce mutations on the X-chromosome–linked targets. X-shredding involves the sgRNA targeting X-linked repeats, resulting in ‘shattering’ of the X chromosome. The sperm carrying the shattered X chromosome cannot produce viable offspring after fertilisation, resulting in all-male offspring. X-meddling involves targeting X-linked haplo-insufficient genes. Therefore, when the knock-out allele containing sperm fertilises the oocyte, the female is nonviable, resulting in single-sex progeny. (c,d) CRISPR/Cas9 bicomponent systems have been generated in the mouse and silkworm. Offspring coinheritance of a Cas9 and sgRNA-transgenic allele targeting an essential viability gene results in mutation and loss of function of the target. Inheritance of a single transgene is predicted to have no effect. (e) Suppression gene drive has been generated in the mouse and mosquito models, amongst others. The CRISPR/Cas9 transgene is targeted to an essential male/female-specific fertility or viability gene in order to disrupt gene function. Expression of the CRISPR-Cas9 transgene converts the transgene from hemizygosity to homozygosity. Loss of function of the target gene renders the target female or male population sterile or nonviable. In order to generate single-sex litters, the CRISPR-Cas9 gene target is a sex determination gene such as *doublesex*, which theoretically would skew sex offspring sex ratios. chr., chromosome; sgRNA, single guide RNA.

A superior system of generating single-sex litters in the laboratory and agriculture would be to produce a single type of sex-chromosome–carrying gamete, i.e., only X- or only Y-carrying sperm, by selective destruction of the unrequired sperm. Evidence for the first genetic methods to skew offspring sex ratios by destruction of the X-carrying sperm was demonstrated in the *Anopheles* mosquito model [89]. *Anopheles* males are heterogametic, XY, whilst females are XX. An I-PpoI (*Physarum polycephalum* intron-encoded endonuclease) cassette was genetically engineered onto the Y chromosome. The I-PpoI endonuclease selectively targeted the X chromosome, resulting in endonuclease-driven shredding of the X [89,90]. The damage to the X-carrying gametes meant that only Y-carrying gametes were able to fertilise oocytes, resulting in a male-biased sex ratio skew [89–92]. Using CRISPR/Cas9, the strategy was refined to target X-linked repetitive ribosomal DNA sequence by single guide RNA (sgRNA)-guided Cas9 endonuclease activity (Fig 1A). Again, the X-shredding resulted in the loss of X-carrying gametes and a male-biased sex skew in offspring, ranging from 86.1% to 94.8% [93]. Most recently, Simoni and colleagues described a successful male-biased distorter system that harnesses a CRISPR-gene drive, inserted into the conserved *doublesex* intron 4–exon 5 boundary, driving I-PpoI to induce X-shredding [94].

Faluso and colleagues expanded on the Galizi and colleagues [90,93] studies by modelling X-shredding and a new strategy called X-meddling (Fig 1B) in *Drosophila* [95]. They produced germline-expressed Cas9 endonuclease lines and bred them with engineered sgRNA-encoding lines, targeting multiple repeat sequences on the X chromosome (for X-shredding) and putative haplo-insufficient genes on the X chromosome (for X-meddling). The authors noted that the majority of X-shredding sgRNAs, bar one, did not substantially affect the progeny sex ratio. Conversely, however, sgRNAs targeting the haplo-insufficiency genes *RpS6* (Ribosomal protein S6) and *RpS5a* (Ribosomal protein S5a) generated a male-biased offspring sex ratio skew from 93.8% to 56.6%, respectively. This study highlighted that although the X-shredding strategy was applicable to other species outside mosquitos, X-meddling proved to be a more efficient method of producing sex-biased progeny and may be more greatly applicable to other species.

### Bicomponent CRISPR/Cas9 systems

Another CRISPR/Cas9-based method for generating single-sex litters is a bicomponent system. A bicomponent CRISPR/Cas9 system refers to the genetic isolation of Cas9- and sgRNA-encoding transgenes. The isolated Cas9 and sgRNA transgenes are coinherited independently of each other from either parent. Integration of either the Cas9 or sgRNA transgene onto a sex chromosome of a heterogametic parent allows for sex-specific inheritance of the transgene. Monoinheritance of either the Cas9 or sgRNA transgene in isolation is predicted to have no mutational effect and therefore is advantageous because monoallelic stocks can be bred normally. Furthermore, it is known that constitutive expression of transgenic Cas9 is not detrimental to mice [96–98]. Conversely, the coinheritance of both transgenes, i.e., one from each parent, would generate loss-of-function mutations at the sgRNA target viability locus, thereby resulting in embryonic lethality of the unrequired sex (Fig 1C and 1D).

The first implementation of a CRISPR/Cas9 bicomponent system to breed all-male offspring was in *B*. *mori* [99]. Similarly to birds, female silkworms are the heterogametic sex (ZW) and males are the homogametic sex (ZZ). Zhang and colleagues generated a female-specific W-linked Cas9 transgenic line, and the transgene was therefore uniquely inherited by daughters [99]. Second transgenic lines were produced carrying an autosomal sgRNA targeting the essential gene *Bmtra2* (*B*. *mori* transformer 2). Coinheritance of the W-Cas9 and sgRNA transgenes in daughters resulted in *Bmtra2* CRISPR/Cas9-induced mutations, 50% of the progeny did not hatch, and the surviving progeny were 100% male. This study was the first to highlight that sex-specific coinheritance of CRISPR/Cas9 components targeting an essential gene produces single-sex litters. Developments in generating single-sex offspring by genetic methods has the potential to be extremely advantageous for the silkworm industry.

In the Zhang and colleagues silkworm study, the aim was to produce all-male progeny. However, laboratories or agricultural applications may require all-female litters; for example, female rodents to study female-specific biology or in producing layer hens (Table 1). In the Zhang and colleagues study, the Cas9 transgene was W-linked and therefore inherited uniquely by females, alongside the autosome-linked sgRNA, to induce *Bmtra2* mutations and female-specific lethality. The same principle is relevant to eutherian mammals; integration of a Cas9 transgene onto the Y chromosome would ensure unique inheritance by sons. Coinheritance of a Y-linked CRISPR/Cas9 component transgene and autosome-linked CRISPR-Cas9 component will induce mutations in the target gene uniquely in sons. If the target gene is an essential viability gene, the sons will be embryonic lethal.

However, the Y chromosome has been extremely challenging to genetically modify because of its highly heterochromatic and repetitive nature. The modern eutherian X and Y chromosomes diverged from a pair of ancestral autosomes [100] following the acquisition of the male-determining gene *SRY/Sry* approximately 148–166 million years ago [101]. Recombination was thereafter suppressed between the X and Y chromosomes, most likely through a series of Y-chromosome inversions [102]. The Y chromosome became recombinationally inert, accumulating deleterious mutations and losing most of its ancestral genes [103]. The clonal inheritance of the Y chromosome through the male lineage contributed to the sexual conflict between the X and Y, leading to further specialisation of Y-genes for male-function [18,103,104] and testis-specific expression [105]. The loss of Y-chromosome genes led to the male-specific Y chromosome being greatly reduced in size compared to the X chromosome. Similarly, in many bird species, the female-specific W chromosome is also greatly reduced compared to the Z chromosome. The comparative sizes of the X and Y or Z and W chromosomes are highly diverse amongst species.

Given the complexity associated with Y-gene targeting, the first study generating a targeted mammalian Y-gene reporter was not published until 2013, using transcription activator-like effector nucleases (TALENs; [106]). Previous attempts to mimic Y-linked transgene expression have utilised Y-gene promoter-driven transgenic lines; however, transgenes are instead randomly integrated into an autosome. For example, an enhanced green fluorescent protein (eGFP) reporter, driven by the *Sry* promoter, was randomly integrated into the genome by zygotic microinjection [107]. The main disadvantage of *Sry*-promoter–driven transgenic alleles, however, is that mouse *Sry* expression is tightly regulated in the gonad, occurring between embryonic day (E) 10.5 and E12.5 [14–16]. Therefore, Cas9 or sgRNA transgene expression would also be limited to the gonad within these developmental time points, and expression would be insufficient to drive sgRNA-guided mutations to produce single-sex litters.

Given that *Sry*-promoter–driven expression is restricted, a preferable choice would be a promoter driving a Y-gene with ubiquitous expression. There are multiple Y-linked genes ubiquitously expressed in the mouse, including *Uty* (Ubiquitously transcribed tetratricopeptide repeat containing, Y-linked) [108], *Eif2s3y* (Eukaryotic translation initiation factor 2 subunit 3, Y-linked) [109], *Ddx3y* (DEAD-Box helicase 3, Y-linked) [110], and *Kdm5d* (Lysine demethylase 5D) [111]. Of these, *Uty* is expressed during embryonic development and also in embryonic stem cells (ESCs) [112]. Furthermore, previous studies have successfully generated in-frame knock-in *Uty*-eGFP reporter ESC lines [106]. Moreover, Zhao and colleagues recently generated a Y-linked reporter mouse line wherein eGFP expression was driven by a constitutive promoter and shown to be expressed in preimplantation embryos [113]. The transgene was inserted into an intergenic region between *Uty* and *Ddx3y*, thereby opening up new possibilities of Y-chromosome knock-in targets.

In 2019, the first mammalian CRISPR/Cas9 bicomponent system was described, with the intention of generating all-female litters by CRISPR/Cas9-induced knock-out of essential genes in male embryos. Yosef and colleagues utilised the ubiquitous Y-linked *Uty* locus to integrate a constitutively expressed sgRNA transgene into the second intron [114], which would therefore be uniquely inherited by sons. Therefore, upon the male-specific coinheritance of the Y-linked sgRNA transgene targeting essential genes *Atp5b* (ATP synthase, H+ transporting mitochondrial F1 complex, beta subunit), *Casp8* (Caspase 8), and *Cdc20* (Cell division cycle protein 20) and an autosomal constitutively expressing Cas9 transgene resulted in knock-out of the target loci and significant male-biased offspring sex ratio [114]. Whether *Uty* expression was affected in these sgRNA-transgenic males was not addressed.

Bicomponent systems are advantageous because mono-transgenic stocks can be maintained as separate lines and bred when necessary. The human control of using original stocks maintained independently ensures that genetic mutational resistance at the target loci is unlikely to occur. Furthermore, if mutational resistance did arise in any offspring, the individuals can be removed from the population without any negative impact on the breeding stocks. Refining the technologies further, it may be possible to generate transgenic laboratory models wherein the transgenes that induce the embryonic lethality effect are inherited by maternal deposition of mRNAs. In an analysis of embryos derived from Gt(ROSA)26Sor (*Rosa26*)-Cas9 hemizygous transgenic mothers, maternally loaded Cas9 was sufficient to induce mutations even in non-transgenic offspring [96].

### Gene drive

Gene drive refers to the process of a selfish genetic element transmitting through a population at above-mendelian frequency [115–117]. Laboratory engineered gene drives can therefore be harnessed to spread genetic traits quickly through a population, and moreover, they can be adapted for producing single-sex progeny. Burt first postulated that endonuclease-driven gene drives could be used for pest-control management [91]; however, the advent of CRISPR/Cas9 has enhanced the potential for synthetic gene drives to be transmitted highly effectively in wild populations [118]. Engineered gene drives function by the insertion of an endonuclease transgene into a target locus. The transgene-encoded endonuclease then copies itself into the other wild-type allele, thereby converting from hemizygosity to homozygosity, and ensuring inheritance by all offspring [91]. ‘Suppression drive’ is a refined engineered gene-drive system, whereby the endonuclease-encoding transgene is inserted into an essential fertility or viability gene [91,118]. Upon active drive and transgene conversion to homozygosity, the individual becomes infertile or nonviable. In CRISPR/Cas9-engineered gene drives, a transgene encoding an sgRNA and Cas9 is integrated into a target viability or sex-specific fertility locus. Transgene expression converts the transgenic allele to homozygosity, thereby rendering individuals nonviable or infertile. These gene-drive methods could be highly efficient for reducing population size. Moreover, the engineered gene drive could be modified to target genes for sex determination. In this strategy, gene-drive–induced modifications of the target locus could produce single-sex progeny (Fig 1E).

In 2018, Kyrou and colleagues generated a gene-drive system in mosquitos, targeting the *doublesex* gene [119]. In this strategy, a gene-drive construct was engineered targeting the female-specific exon of *doublesex*, leaving the male *doublesex* splice variant unaffected, aiming to produce male-biased progeny. Heterozygous targeted females were unaffected, confirming that *doublesex* is functional with a single copy (haplo-sufficient). Interestingly, homozygous targeted females were not sex reversed but instead showed an intersex phenotype and were infertile [119]. This study highlights that currently gene drives cannot be used for generating sex-biased litters but instead could be used to cause a population collapse by sex-specific sterility.

To examine whether the synthetic suppressive drive systems could also be applied to mammals, Grunwald and colleagues performed the first proof-of-principle gene-drive system in mice [120]. To assess success, they utilised the *Tyrosinase* (*Tyr*) gene, which generates white-coated mice upon homozygous knock-out. An sgRNA transgene targeting *Tyr* and also encoding an mCherry reporter was inserted into the *Tyr* locus to produce a *Tyr*-heterozygous knock-out and hemizygous transgenic mouse line. When bred with Cas9-expressing mice, functional gene-drive systems would transmit to offspring at above-mendelian frequency. A successful gene drive should produce *Tyr* loss-of-function white mice that also express mCherry. In this approach, gene-drive success varied from 0% to 72%. Often, the mice were white-coated but did not express mCherry, suggesting that transmission of the sgRNA and Cas9 transgenes produced mutations at *Tyr* but without copying the sgRNA/mCherry transgene [120]. The repair pathway after CRISPR/Cas9-induced mutations at *Tyr* was likely nonhomologous end-joining (NHEJ), consistent with previous reports that NHEJ is the dominant mode of repair over homology-directed repair [121,122]. Therefore, although relatively efficient in mosquitos [123], mammalian synthetic gene drives require further optimisation.

## Disadvantages of genetic methods

Genetic or genome editing systems have the potential to effectively generate single-sex litters; however, they currently have some disadvantages. The superior method of generating single-sex litters is by selective destruction of the nonrequired sperm. One method to selectively destroy X-carrying sperm is by X-shredding or X-meddling. Although this technique was shown to be highly efficient in mosquitos [90,93], it was variable in *Drosophila* [95]. An important consideration of harnessing X-shredding in other nonmosquito species is the availability of X-targets because this is likely to strongly influence the success rate. One alternative method is by targeting the X-shredding transgene to the Y chromosome for germline expression. The question of whether the Y chromosome could be used for *Drosophila* CRISPR/Cas9 transgene expression for X-meddling or X-shredding is still open; however, great strides have been made in determining possible transgene integration sites [124].

Although CRISPR/Cas9 bicomponent systems are advantageous in that the mono-transgenic stocks can be easily maintained independently, current bicomponent systems are not 100% efficient. In the Yosef and colleagues study [114], the sex skew was imperfect; i.e., some males were born despite mutations in the target housekeeping genes. Furthermore, these males were often born with severe developmental abnormalities, which raises further ethical questions in line with the 3Rs. Another interesting question regarding bicomponent systems is that of the number of offspring born. The bicomponent CRISPR/Cas9 system selectively induces nonviability in a target sex by CRISPR/Cas9-induced mutations in a target gene. Therefore, by estimates of mendelian frequency, approximately half of the offspring are embryonic lethal. Although the unrequired sex is not born, the number of pups of the required sex remains unchanged. Moreover, there is potentially a risk that the in utero embryonic lethality of the unrequired sex could also stimulate abortion of the required sex. As earlier described, a superior method to generating single-sex litters would be via selective destruction of the unrequired sex-chromosome–carrying sperm, such as by X-shredding. In this strategy, the surviving sperm are free to fertilise all available oocytes, and therefore, all of the offspring that are born are of the required sex.

One possible disadvantage of a released gene-drive genetic system is through mutational resistance arising at the sgRNA target site. If a nucleotide variant arises at the sgRNA target or the neighbouring protospacer-adjacent motif (PAM), then the CRISPR/Cas9 system becomes immediately dysfunctional. Mutational resistance to an embryonic-lethal gene drive could potentially spread efficiently through the population because of conferring a fitness advantage. Indeed, evidence for rapidly arising resistance alleles has been shown recently in flies [125] and mosquitos [126]. Current studies suggest that prevention of newly arising resistance mutations at gene-drive target loci would therefore require continuous intervention [91,127,128]. However, in laboratory or factory-maintained stocks, the issue of mutational resistance would not occur because these individuals could be simply removed from the population. One possible circumvention of the mutational resistance risk is to engineer many sgRNA transgenes targeting multiple loci. Therefore, even if mutational resistance occurs at one locus, the remainder are intact.

A second disadvantage associated with CRISPR/Cas9 gene drive is the risk that the above-mendelian frequency of inheritance spreads so rapidly through the population that the original wild-type allele is completely lost. The complete loss of the wild-type allele may cause apprehension against gene drive. Adaptations to gene-drive methods have been developed in order to prevent the uncontrolled spread of synthetic gene drives, called ‘self-exhausting’ gene drives, for example, the killer-rescue [129] or daisy-chain [130] models.

## Summary and future outlook

Overall, there are many challenges still associated with producing transgenic mouse lines in order to produce single-sex litters. However, with careful consideration of which genes are targeted to induce embryonic lethality or sterility, bicomponent CRISPR/Cas9 methods could be widely employed to skew sex ratios. For example, targeting genes that are essential in postimplantation development may not result in a complete loss of the unrequired sex. Instead, an alternative approach may be to target essential housekeeping genes with roles in preimplantation development at embryonic genome activation. Embryonic lethality can then be induced very early, before the onset of organogenesis. Inducing embryonic lethality at preimplantation ensures firstly that embryos are nonviable prior to the onset of pain perception. Secondly, it would be interesting to determine whether embryo loss due to nonviability prior to implantation may allow extra viable embryos to implant, thereby compensating the litter size.

Even if all of the necessary optimisations are made and the technology for generating single-sex litters is consistent, there may be some apprehension regarding using genetically modified animal produce in agricultural industries. Utilising maternally loaded mRNAs such as the earlier described *Rosa26*-Cas9 may circumvent issues with genetically modified offspring for agricultural produce. Moreover, it may be that some consumers consider the use of transgenic animals in agriculture ethically preferable to the widespread culling of the unrequired sex. Indeed, there are some examples of genetically modified produce currently in the food industry, for example, a modified salmon species with increased growth rate [131], although it should be made clear that many regulatory limitations remain in place regarding the sale and consumption of genetically modified animal produce. It is important to continue the global conversation regarding the role of genetic modification in the agricultural industries. However, in the short term, it is more likely that the sex-selection strategies could be quickly and easily implemented for immediate reduction of postnatal animal culling in laboratory animals such as mice in line with the 3Rs.
